# Conspecific plants are better ‘nurses’ than rocks: consistent results revealing intraspecific facilitation as a process that promotes establishment in a hyper-arid environment

**DOI:** 10.1093/aobpla/plx056

**Published:** 2017-10-24

**Authors:** Andrea P Loayza, Marisol A Herrera-Madariaga, Danny E Carvajal, Patricio García-Guzmán, Francisco A Squeo

**Affiliations:** Department of Biology, University of La Serena, Av. Raúl Bitrán 1305, La Serena 1720256, Chile; Institute of Ecology and Biodiversity (IEB), Las Palmeras 3425, Ñuñoa, Santiago 7800003, Chile; Center for Advanced Studies in Arid Zones (CEAZA), Av. Raúl Bitrán 1305, La Serena 1720256, Chile

**Keywords:** Arid ecosystems, germination, intraspecific facilitation, nurse rocks, plant regeneration, seed predation, seedling establishment

## Abstract

Harsh environmental conditions in arid ecosystems limit seedling recruitment to microhabitats under nurse structures, such as shrubs or rocks. These structures, however, do not necessarily afford the same benefits to plants because nurse rocks provide only physical nurse effects, whereas nurse plants can provide both physical and biological nurse effects. Nevertheless, if the nurse plant is a conspecific, the benefits it provides may be outweighed by higher mortality due to negative density-dependent processes; consequently, negative density-dependence is expected to limit plants from acting as nurses to their own seedlings. The degree to which an abiotic nurse may be more beneficial than a conspecific one remains largely unexplored. Here, we examine the role and elucidate the mechanisms by which conspecific plants and rocks promote plant establishment in a hyper-arid desert. For 4 years, we examined establishment patterns of *Myrcianthes coquimbensis* (Myrtaceae), a threatened desert shrub that recruits solely in rock cavities and under conspecific shrubs. Specifically, we characterized these microhabitats, as well as open interspaces for comparison, and conducted germination, seed removal and seedling survival experiments. Our results revealed that conspecific shrubs and nurse rocks modified environmental conditions in similar ways; soil and air temperatures were lower, and water availability was higher than in open interspaces. We found no evidence on negative density-dependent recruitment: seed removal was lowest and seedling emergence highest under conspecific plants, moreover seedling survival probabilities were similar in rock cavities and under conspecific plants. We conclude that the probability of establishment was highest under conspecific plants than in other microhabitats, contrasting what is expected under the Janzen–Connell recruitment model. We suggest that for species living in stressful environments, population regulation may be a function of positive density-dependence and intraspecific facilitation may be a process that promotes the persistence of some plant species within a community.

## Teaser

In stressful environments, nurse elements, such as shrubs or rocks, facilitate plant recruitment by providing less severe environments for seed germination and seedling survival. As seedlings develop, however, they may compete for resources with their nurse when it is a plant instead of a rock. We examined the role of conspecific plants and rocks as nurses of an endangered Atacama-Desert shrub. Establishment was highest under conspecific plants, revealing that in stressful environments plants can facilitate establishment of their own species. Our study contrasts findings from other environments where recruitment is lowest near plants of the same species.

## Introduction

It is widely recognized that in desert environments plant regeneration via seedling recruitment is rare and sporadic (Squeo *et al.* 2007). In these ecosystems, high temperatures and low soil water availability lead to low germination and seedling survival rates (Flores and Jurado 2003; Martinez-Tilleria *et al.* 2012). Therefore, recruitment in arid-lands is generally restricted to habitats or microsites where more benign conditions prevail (Flores and Jurado 2003; Carvajal *et al.* 2014). Typically, the set of environmental conditions that allows seeds to germinate and become established (i.e. the recruitment or establishment niche; Young *et al.* 2005) are found beneath the canopies of adult plants or within rock crevices; hence, seedling recruitment in arid environments is frequently associated to these elements, which are referred to as ‘nurse plants’ and ‘nurse rocks’, respectively (Shreve 1931; Wied and Galen 1998; Flores and Jurado 2003; Munguia-Rosas and Sosa 2008; Peters *et al.* 2008). These nurse elements provide seedlings with a more benign microclimate, as well as with improved soil water and nutrient availability (Callaway 1995).

While both nurse plants and nurse rocks can ameliorate the abiotic stress exerted by the environment, these structures do not necessarily afford the same benefits to plants. For example, nurse rocks do not ameliorate soil conditions, whereas the soils under nurse plants typically have a higher content of organic matter, nitrogen and potassium than open areas, which may further facilitate germination and growth (Callaway 1995; Pugnaire *et al.* 2004; Molina-Montenegro *et al.* 2016). On the other hand, seedlings sheltered by nurse rocks do not compete with their nurse at any stage during their ontogeny; in contrast, seedlings under nurse plants may compete with their nurse as they grow and their resource requirements change (i.e. ontogenetic niche shifts) resulting eventually in lower growth and/or survival of either partner (Miriti 2006).

Competition between a plant and its nurse can occur irrespective of the identity of the nurse, but it is predicted to be stronger among plants of the same species (i.e. conspecific plants) than among those of different species (i.e. heterospecific plants), because the former have similar niche requirements (Janzen 1970; Silvertown and Charlesworth 2009; Bonanomi *et al.* 2010). Additionally, mortality is expected to be higher under conspecific than heterospecific plants due to increased rates of detection by predators (Janzen 1970; Connell 1971). Therefore, negative density-dependent processes are ultimately expected to limit plants from acting as nurses to seedlings of their own species (Bonanomi *et al.* 2010).

Nevertheless, and in spite of being rare, facilitation among conspecific plants does occur and is referred to as ‘intraspecific’ facilitation (Wied and Galen 1998; Weltzin and McPherson 1999; Eränen and Kozlov 2008; Cavieres and Penaloza 2012). Intraspecific facilitation has been reported especially in highly stressful environments (Eränen and Kozlov 2008; Goldenheim *et al.* 2008; Fajardo and McIntire 2011) because to a certain extent, the benefits gained by heterospecific nurses can also be gained by conspecific ones. Moreover, when intraspecific facilitation occurs, it can lead to individuals forming dense aggregations in which they still compete with each other for resources, but where these costs do not outweigh the benefits of group living (Stachowicz 2001; Goldenheim *et al.* 2008).

**Figure 1.**
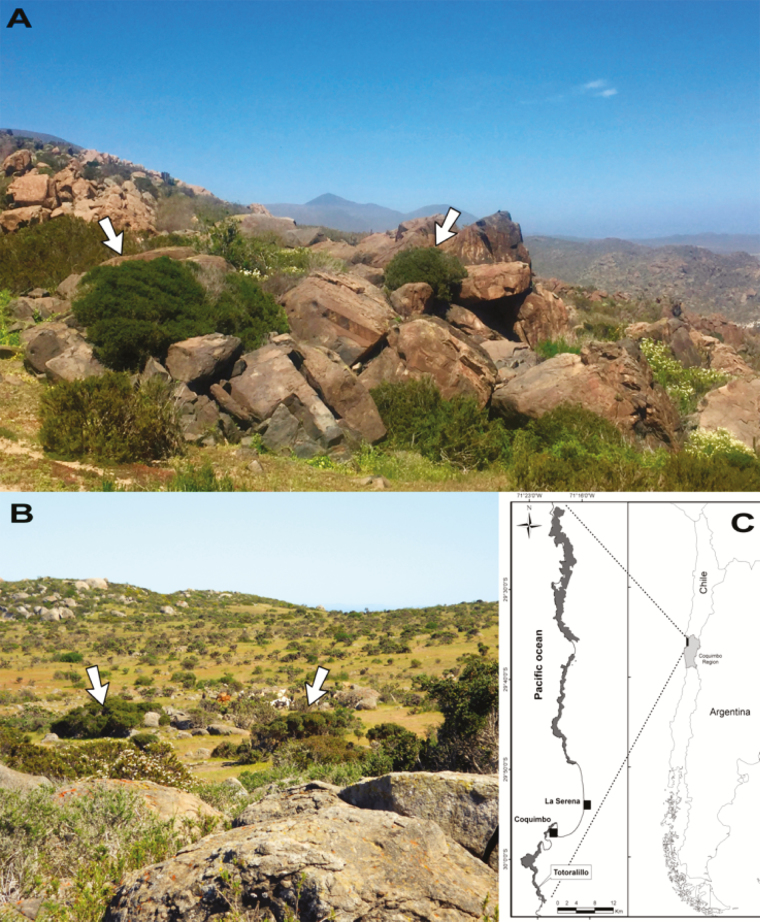
(A) Adult *Myrcianthes coquimbensis* shrubs growing from rock cavities; native rodents bury and/or hoard seeds into these cavities. (B) A typical landscape where *M. coquimbesis* is found; the arrows point to two *M. coquimbesis* monospecific stands. (C) Map showing the distribution (dark grey) of *M. coquimbensis*. The study site (Totoralillo) is located in the southern edge of its distribution. All photos were taken after the onset of the winter rains.

Thus far, most studies examining nurse–protégé interactions have focused on the facilitative effects of heterospecific nurses, while quantitative data on the role of other nurse elements, such as rocks or conspecific plants, are still scarce. Our study aims to examine the role and elucidate the mechanisms by which conspecific nurse plants and nurse rocks may promote plant establishment. To this end, we selected *Myrcianthes coquimbensis*, an endangered Atacama Desert shrub. This species has recalcitrant seeds that do not enter the seed bank, but desiccate and die within a few weeks if not sheltered from solar radiation. Accordingly, seedlings—which are extremely rare (i.e. <0.02 % of the total population)—are found exclusively in sheltered microhabitats; they are not present in open interspaces (García-Guzmán *et al.* 2012). However, seedlings are only found in two types of sheltered microhabitats: (i) within crevices in rock outcrops (rock cavities hereafter) and (ii) under conspecific plants; no seedlings have been recorded growing under the canopies of other shrub species (García-Guzmán *et al.* 2012). The pattern of adult distribution is to some degree concordant with that of seedlings: most plants grow either individually from rock cavities (Fig. 1A) or forming small monospecific patches in open interspaces (Fig. 1B). Yet, in contrast to seedlings, a small percentage of the adults (<30 %) also grow isolated in open interspaces (unpubl. data). Plant establishment in open areas was likely more feasible in the past, because annual rainfall was at least double of what it is today (see Methods).

Together, the patterns of seedling and adult distribution suggest that both adult conspecific plants and rocks promote *M. coquimbensis* establishment; however, the degree to which an abiotic nurse may be more beneficial than a conspecific one remains unexplored. While a nurse rock will only provide a physical nurse effect, a conspecific plant may provide both physical and biological nurse effects, yet the benefits provided by conspecific nurses may be outweighed by higher mortality due to negative density-dependent processes. Hence, in this study, we addressed the following questions: (i) How do nurse rocks and conspecific nurse plants modify air and soil environmental conditions? (ii) How do these nurse elements influence emergence, seed and seedling survival? (iii) What is the net outcome between facilitative and negative effects on the mid-term (i.e. 4 years) probability of establishment? We predicted that both conspecific plants and nurse rocks would ameliorate environmental conditions in similar ways, but that soil nutrient content would be higher under conspecific nurses. As a consequence, we expected similar emergence rates under conspecifics and in rock cavities. However, we predicted higher seed and seedling mortality under conspecifics than in rock cavities due to negative density-dependence, thus ultimately, we hypothesized that the probability of establishment would be higher in rock cavities than under conspecific plants.

## Methods

### Study area

We conducted this study in Totoralillo (30°04′S, 71°14′O; 0–33 m a.s.l.), which is located 26 km south of La Serena (Chile). The area is in the southern limit of the Atacama Desert; it is a hyper-arid environment (<60–100 mm mean annual precipitation; Noy-Meir 1973) characterized by having a coastal foggy climate with a mean annual temperature of 14 °C. Rainfall is extremely variable, with rainy years associated to ENSO events (Squeo *et al.* 2006). In spite of this variability, however, annual precipitation in the area has gradually declined from a mean of 170 mm per year at the beginning of the 20th century, to *ca.* 80 mm per year during the past 10 years (79.4 ± 32.4, mean ± SD; CEAZAMET). Rain can fall from March to November, but pulses typically occur between May and September.

In addition to *M. coquimbensis*, the dominant plant species in this coastal desert are *Oxalis gigantea*, *Heliotropium stenophyllum* and *Fuchsia lycioides*, along with cacti, *Eulychnia breviflora*, *Echinopsis coquimbana* and *Copiapoa coquimbana*. Note that, with the exception of *H. stenophyllum*, none of these species have canopies that provide shade and modify the microenvironmental conditions beneath them so as to act as nurse plants. Woody vegetation covers between 25 and 30 % of the ground; the rest is generally bare except for the presence of rock outcrops composed of large boulders, in between which are cavities with exposed soil where plants can recruit.

### Study species

*Myrcianthes coquimbensis* (Myrtaceae) is a narrow endemic shrub restricted to 83 km^2^ along the coast of Chile’s Elqui Province (Fig. 1C) (García-Guzmán *et al.* 2012). This species can flower from October to March, but the peak bloom period usually occurs between November and December. *Myrcianthes coquimbensis* has large, fleshy drupes that are available from July through December (Peña 2016); the peak of the fruiting season, however, occurs in October. Because fruits take almost a year to develop and mature, plants can simultaneously bear ripe fruits that developed from the flowers of the previous year and flowers from the current year. Fruits generally contain only one recalcitrant seed (Luna 2016), which desiccates within a month. Native rodents collect ripe fruits that have fallen under the parent plant and hoard or bury them in (i) rock cavities, (ii) under other *M. coquimbensis* plants and less frequently, in (iii) open interspaces (Loayza *et al.* 2014; Luna 2016).

### Microhabitat characterization

We selected three microhabitats to conduct the experiments: (i) open interspaces, which have little or no vegetation cover and are exposed throughout the day to high solar radiation; (ii) under conspecific shrubs, which is a shady microhabitat and its soil is covered with leaf litter; and (iii) rock cavities, which are characterized by having very low solar radiation and no leaf litter.

To characterize abiotic conditions in each microhabitat, we measured soil and air temperatures, soil water potential (ψ_s_) and gravimetric water content (GWC) in two sampling periods: March and September 2014. Additionally, for samples taken in March 2014, we quantified soil nutrient content and generated soil water retention curves (WRCs) from which we calculated field water capacity (FC) and the permanent wilting point (PWP). Typically, March is the driest month during the year; therefore, it represents the time period with the most stressful environmental conditions. In contrast, in September soils are generally moist following the winter rainfall. All measurements were made between 0800 and 1000 hours. Both soil and air temperatures were measured for 25 replicates per microhabitat using a thermocouple (CHY 500 type K); soil temperature was recorded at 3 cm depth, and air temperature at 10 cm above the ground. We measured ψ_s_ and GWC in 10 replicates per microhabitat. For these analyses, soil samples were obtained at a depth of 20 cm, placed in sealed plastic bags and transported in a cooler to the laboratory for processing within a 3-h period. We measured ψ_s_ with a WP4 Dewpoint PotentiaMeter (Decagon Devices, Pullman, WA, USA). To calculate GWC, we weighed ~30 g from each soil sample using a scale (Scaltec SBC 31 electronic balance with a readability of 0.0001 g) and then oven-dried (Binder FED 53–720) samples at 70 °C for 48 h. We calculated GWC as: (FW − DW/DW) * 100, where FW and DW are the fresh and dry weight, respectively, of each soil sample. We examined if soils from different microhabitats differed in their water storage capabilities by generating WRCs from five soil samples of each microhabitat **[see Supporting Information—Appendix S1]**. Finally, 5 soil samples per microhabitat were measured using standard soil analytic methods at the Centro Tecnológico de Suelos y Cultivos of Universidad de Talca (Chile) to determine whether the soil samples from each microhabitat differed in their nitrogen (N), phosphorous (P), potassium (K) and organic content (OC), as well as in their conductivity (C) and pH.

### Seed removal and seedling emergence experiments

Seed removal and seedling emergence were monitored during three fruiting seasons (2011, 2012 and 2013). In all years, experiments were established in September.

To determine whether seed removal rates vary among habitats, in 2011 and 2012, we set up experimental seed depots consisting of 10 *M. coquimbensis* seeds in each of 10 replicates of two microhabitats: rock cavities and under conspecific shrubs. During those 2 years, we did not establish seed removal experiments in open interspaces because: (i) in arid environments rodents typically forage in sheltered environments (Simonetti 1989; Meserve *et al.* 1993; Lagos *et al.* 1995), and (ii) fruits and seeds of *M. coquimbensis* accumulate in large quantities only under conspecifics and in rock cavities. Nonetheless, in 2013, we found evidence of rodents caching seeds in open areas, thus we set up 10 seed depots of 10 seeds each, in each of the three microhabitats. In all years, we recorded the final number of removed seeds 3 weeks after setting up the experiment. Note that we used seed removal as a proxy of seed predation because rodents remove seeds and store them for later consumption; however, some of these seeds may be forgotten and hence dispersed. Therefore, although a removed seed will most likely be consumed, it does not necessarily equate a predated seed.

We determined if the rate of seedling emergence differs among open interspaces, rock cavities and under conspecific shrubs by sowing a group of 10 seeds in each microhabitat replicate (*N* = 10 microhabitat^–1^ year^–1^). In each replicate, seeds were placed partially buried in the ground, and each group was protected by a 25 × 20 × 10 wire cage (5 * 5 mm mesh size) to prevent rodents from removing seeds. Seedling emergence was monitored once a month for 6 months.

### Seedling survival

We assessed whether seedling survival differs among microhabitats by comparing the establishment probability of all seedlings that emerged from the emergence experiments described above. Once seedlings emerged they were individually marked with an aluminium tag and their fate was followed for 4 years.

### Probability of recruitment

We examined establishment probabilities vary among microhabitats via the estimation of transition probabilities (TPs) between different plant stages in each microhabitat. Transition probabilities were estimated as the mean number of individuals completing a stage divided by the number of individuals entering that stage (Rey and Alcantara 2000; Loayza *et al.* 2011). Finally, the probability of recruitment at each microhabitat was calculated as the product of the individual TPs.

### Statistical analyses

We evaluated variation in abiotic conditions among microhabitats by means of generalized linear models (GLMs) considering microhabitat as the main factor, and temperature (soil and air), ψ_s_, GWC, FC and PWP as the response variables. We examined whether soils of different microhabitats differed in their chemical characteristics using a permutational MANOVA. This analysis uses permutations to make it distribution free and works with any distance measure that is appropriate to the data. An important assumption of the analysis, however, is that the multivariate spread does not differ among groups, which in our case did not.

Differences in seed predation and seedling emergence among microhabitats were determined using GLMs with Poisson error distributions (link = ‘log’); for these analyses, microhabitat and year (for seedling emergence only) were the main factors, and number of predated seeds and emerged seedlings the response variables. We assessed whether the temporal patterns of seedling mortality varied among microhabitats with Cox’s proportional hazards models that were clustered by habitat replicate to account for non-independence. In these models, comparisons were made against the fate of seeds under conspecific shrubs, as this is the microhabitat where most of the seeds remain. Finally, to determine whether the probability of recruitment differed among habitats we used permutation *t*-tests. The latter were based on 999 permutations of the data. All statistical analyses were performed using the R statistical environment (R Development Core Team 2013; R Foundation for Statistical Computing, Vienna, Austria).

## Results

### Microhabitat characterization

**Figure 2.**
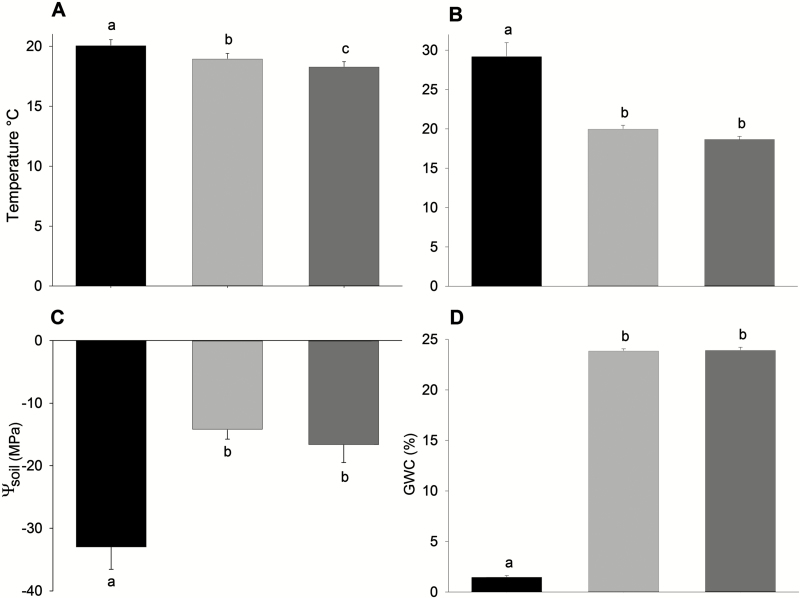
Abiotic conditions in each microhabitat: open interspaces (black), under conspecific shrubs (dark grey) and rock cavities (light grey). (A) Air temperature. (B) Soil temperature. (C) Soil water potential. (D) Gravimetric soil water content. Lowercase letters indicate differences among groups. Means_(March and September)_ ± 1 SE.

Air and soil temperatures varied across microhabitats and between seasons (Table 1). Air temperature differed among all three microhabitats; it was highest in open interspaces and lowest in rock cavities (Fig. 2A). There were no differences in soil temperatures between rock cavities and under conspecific shrubs; however, temperatures in these two microhabitats were significantly lower than in open interspaces (Fig. 2B). We also found significant differences in ψ_s_ and GWC among microhabitats and between seasons (Table 1); specifically, soils in open interspaces had more negative values of ψ_s_ and lower values of GWC than under conspecific shrubs or rock cavities (Fig. 2C and D).

**Table 1.** Summary of the GLM results for differences in abiotic conditions among microhabitats and between seasons. Seasons correspond to late summer and spring of 2014 (March and September, respectively). Significance was assessed using likelihood ratio tests.

Variable	Factor	df	Deviance	Residual deviance	Pr(>Chi)
Air temperature (°C)	Microhabitat	2	80.66	1680.5	<0.0001
	Season	1	1448.65	231.6	<0.0001
Soil temperature (°C)	Microhabitat	2	3295.0	8720.9	<0.0001
	Season	1	5201.5	3519.4	<0.0001
Soil water potential (ψ_s_)	Microhabitat	2	4172.3	8949.9	<0.0001
	Season	1	4822.6	4118.2	<0.0001
Gravimetric water content	Microhabitat	2	11.9	64.81	0.004
	Season	1	7.11	57.69	0.009

There were no differences among habitats in either the soil field capacity (*F*_2, 5_ = 1.00, *P* = 0.40) **[see Supporting Information—Fig. S1]** or the PWP (*F*_2, 5_ = 0.43, *P* = 0.66), revealing that the capacity to retain water does not differ among microhabitats. Soils differed in their chemical characteristics (*F*_model_ = 3.83, *r*^2^ = 0.39, *P* = 0.04, based on 1000 permutations). In particular, soils under conspecific shrubs had higher OC than the other two microhabitats **[see Supporting Information—Fig. S2]**.

### Seed removal and seedling emergence

**Figure 3.**
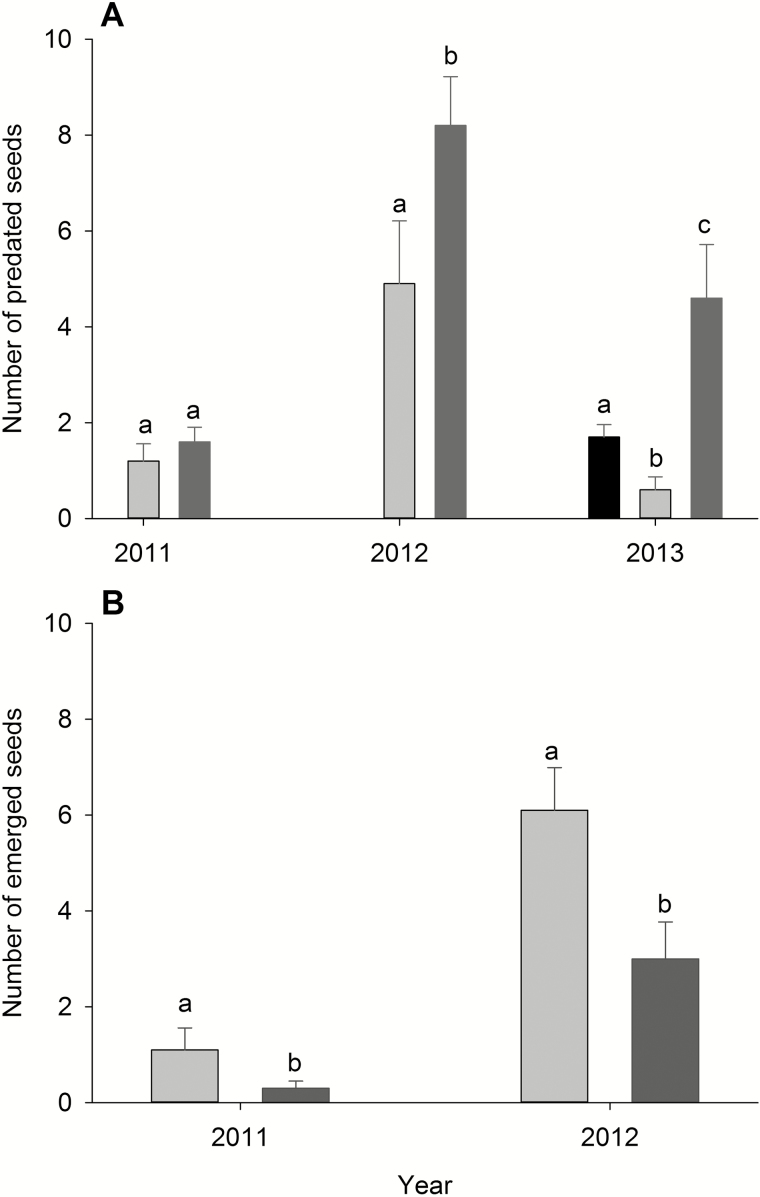
(A) Number of predated seeds after 3 weeks in: open interspaces (black), under conspecific shrubs (dark grey) and rock cavities (light grey). (B) Number of emerged seedlings after 6 months under conspecific shrubs (dark grey) and rock cavities (light grey). For both panels, lowercase letters indicate differences among groups within a single year. Means ± 1 SE.

Seed removal varied among microhabitats in 2012 and 2013, but not in 2011 (Table 2). In 2012 and 2013, it was consistently higher in rock cavities than in the other two microhabitats (Fig. 3A). No seedlings emerged from the 2013 cohort; additionally, no seedlings emerged in open interspaces in any of the years. Therefore, we excluded both the 2013 cohort and open microhabitats from the analyses. The number of seedlings that emerged varied between years and among habitats (Table 3). Seedling emergence was higher under conspecific shrubs than in rock cavities and higher in 2012 than 2011 (Fig. 3B).

**Table 2.** Summary of the GLM results for differences in seed predation among microhabitats from 2011 to 2013. Significance was assessed using likelihood ratio tests.

Year	df	Deviance	Residual deviance	Pr(>Chi)
2011	1	0.57	18.04	0.45
2012	1	8.40	62.22	0.004
2013	2	37.37	44.45	<0.0001

**Table 3.** Summary of the GLM results for differences in seedling emergence among microhabitats and between years. Significance was assessed using likelihood ratio tests.

Factor	df	Deviance	Residual deviance	Pr(>Chi)
Microhabitat	1	18.83	131.62	0.0001
Year	1	63.10	68.52	<0.0001
Microhabitat * Year	1	0.80	67.73	0.37

### Seedling establishment

From 2011 to 2016, there were no differences in the survival probability of seedlings that emerged in rock cavities and under conspecific shrubs (Wald χ^2^ = 0.08, df = 1, *P* = 0.78); at the end of the study, 12 % of the seedlings that emerged under conspecific shrubs and *ca.* 10 % of those that emerged in rock cavities were still alive. During the study period, median survival time for seedlings in both microhabitats was 2 years.

### Probability of recruitment

**Figure 4.**
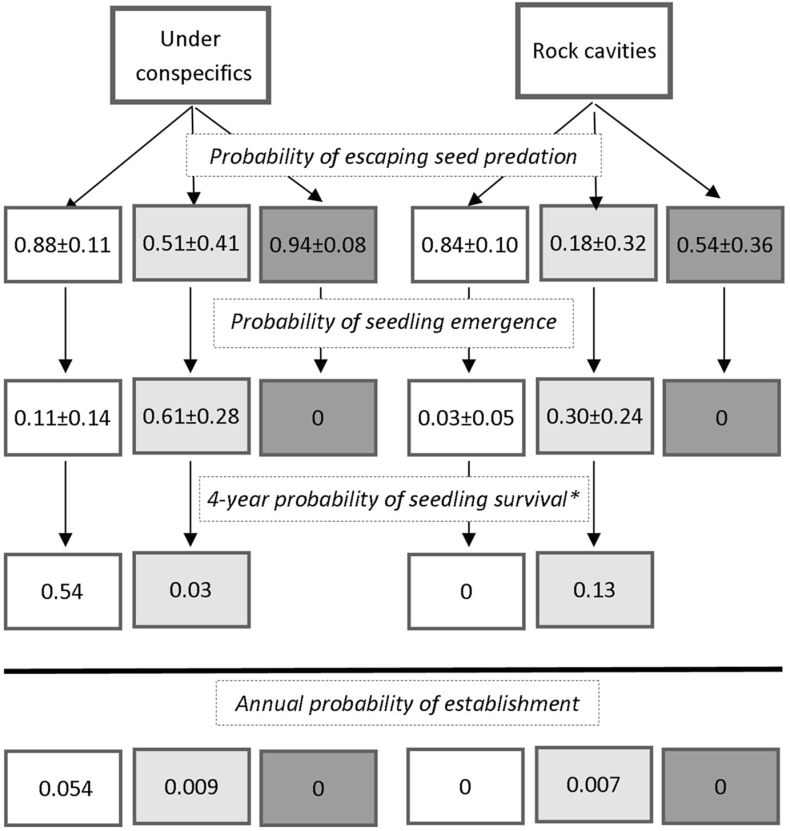
Seed-fate diagram of *Myrcianthes coquimbensis* recruitment in rock cavities and under conspecific shrubs. Each column reflects recruitment for one of the three cohorts (2001, 2012 and 2013). The values represent transition probabilities estimated from the experiments.

Mid-term recruitment probabilities of *M. coquimbensis* in each microhabitat are summarized in Fig. 4. The results reveal that for both the 2011 (*t* = 2.27, *P* = 0.018, permutations = 999) and 2012 (*t* = 2.19, *P* = 0.019, permutations = 999) cohorts, the 4-year probability of establishment was more likely under conspecifics than in rock cavities, and null in open interspaces.

## Discussion

Results from this study can be summarized in two key findings. First, conspecific nurse plants and nurse rocks modify environmental conditions in similar ways; both elements contribute to decrease soil and air temperatures, as well as increase soil water availability. Moreover, in spite of organic matter content being higher under conspecific shrubs, soil nutrient contents are similar beneath both nurse structures. Second, negative density-dependent processes do not appear to limit recruitment because, contrary to what we expected, seed removal was lowest and seedling emergence highest under conspecific plants; additionally, 4-year seedling survival probabilities did not differ between rock cavities and conspecific plants. Overall, our results suggest plant parental care; adult *M. coquimbensis* plants ameliorate the harsh environmental conditions of this hyper-arid environment, which promotes recruitment and ultimately compensates for expected negative density-dependent processes when growing at high densities.

### How do nurse rocks and conspecific nurse plants influence emergence, seed and seedling survival?

We predicted that both nurse elements would modify environmental conditions similarly, which would result in comparable emergence rates under conspecifics and in rock cavities. Our results, however, revealed that even though environmental conditions—in particular soil moisture and water retention capabilities—are in fact similar in both nurse elements, more seedlings emerged under conspecifics than in rock cavities. Consequently, differences in emergence cannot be explained by soil water availability. Soils in these microhabitats did differ in their organic matter content, which was significantly higher under conspecific shrubs probably as a result of falling leaf litter. Leaf litter can increase seed longevity by reducing the rate of seed desiccation (Facelli and Pickett 1991; Molofsky and Augspurger 1992; García *et al.* 2002; Rotundo and Aguiar 2005). For example, Joet *et al.* (2013) found that leaf litter prevented seed drying and subsequent loss of viability of recalcitrant *Quercus* seeds. Moreover, under water-limited conditions, leaf litter presence can have a positive effect on seedling emergence because it maintains soil moisture and air humidity beneath the litter layer and reduces temperature fluctuations (Eckstein and Donath 2005; Loydi *et al.* 2013). Therefore, in the hyper-arid conditions of the Atacama Desert, conspecific leaf litter may provide *M. coquimbensis* seeds with longer a time window to germinate and establish.

In contrast to what we expected, seed predation (i.e. removal) was higher under conspecific shrubs that in rock cavities, suggesting an apparent lack of density-dependent mortality. Seed predation is expected to be heavier near the parent plant because when seeds accumulate under it, their presence may be advertised by the presence of flowers or fruits on the parent, which results in an increased detection rate by predators (Janzen 1970; Connell 1971). In arid environments, the same principle holds true, but the harsh environmental conditions, coupled with the open nature of the habitat, can impose restrictions on the extent to which fruiting plants are visited. For rodents in particular, which are important desert granivores and the only seed predators in our system, the openness of the environment can limit how they move and forage among food patches. In the case of *M. coquimbensis* specifically, the three rodent species that forage on its fruits and seeds avoid moving across open areas (Simonetti 1989; Meserve *et al.* 1993; Lagos *et al.* 1995) because they use light levels as cues of predation risk (Vásquez 1994, 1996) and because of their low heat tolerance (Kenagy *et al.* 2004). Therefore, these species may be more likely to move within rock outcrops to reach food patches, which would ultimately result in a higher seed encounter rate in this microhabitat. Similar results have been reported for *Helleborus foetidus*, where plants in rocky substrates experienced more fruit predation by mice than those growing on mostly bare ground (Fedriani 2005). Additionally, it is also possible that rodents search this microhabitat more frequently than adult shrubs because the rodents that consume *M. coquimbensis* fruits and seeds generally cache them in rock cavities (Luna *et al.* 2016); thus, this strategy would maximize seed pilfering. Seed removal varied among microhabitats in 2012 and 2013, but not in 2011. This is probably because there was very little fruit production on that year (Peña 2016), which may have led rodents to harvest all seeds available in each microhabitat. Similar results were observed by Li and Zhang (2007), who found that seed removal by rodents increased as seed availability decreased. This result highlights the context-dependence of species interactions (Bronstein 1994) showing how variation in biotic and abiotic conditions can alter their outcome.

We did not detect higher mortality of field-emerged seedlings under conspecific shrubs than in rock cavities. In arid environments, water is considered the main constraint for seedling survival and establishment (Squeo *et al.* 2007; León *et al.* 2011); therefore, we expected higher seedling mortality under conspecific shrubs due to strong intraspecific competition for soil water. Our results, however, suggest that there is low competition for water between seedlings and adults, because strong competition for resources would necessarily lead to the detrimental performance of seedlings under conspecific plants (Keddy 2001; Christensen 2007). In addition to water, shade can also influence seedling survival in water-limited conditions. For example, Carvajal *et al.* (2014) showed that seedling survival of four Atacama Desert shrubs is highest in intermediate shade levels and lowest in both low and deep shade environments. Thus, differences in seedling survival under conspecific plants and in rock cavities may relate to the amount of shade provided by each microhabitat. Under deep shade seedlings may not be able to maintain a positive carbon balance (Walters and Reich 1999; Holmgren *et al.* 2012), which would ultimately affect plant survival (Baltzer and Thomas 2007). In this study, we did not characterize the level of shade in each microhabitat, but rock cavities are visually darker than the areas under shrubs.

### What is the net outcome between facilitative and negative effects on the mid-term (i.e. 4 years) probability of establishment?

As with earlier plant stages, the mid-term probability of establishment was higher under conspecifics than in other habitats, revealing that intraspecific facilitation is an important process in promoting the establishment of *M. coquimbensis* shrubs. Traditionally, intraspecific facilitation has been ignored or regarded as a negligible process in structuring vegetation communities (Malkinson and Jeltsch 2007). Consequently, studies examining facilitation have centred on heterospecific nurse effects, and less so on examining the role of intraspecific facilitation on seedling recruitment (Bonanomi *et al.* 2010). This bias may be because there is an implicit assumption that recruitment in most plant populations follows the basic Janzen–Connell model (Janzen 1970; Connell 1971). Recruitment in this model is characterized by a reduction in mortality as distance to the parent (or a conspecific) plant increases, which results from greater competition for resources and/or higher detection rate by consumers; that is, from negative density-dependence processes. In stressful environments, however, population regulation may be a function of positive density-dependence (Goldenheim *et al.* 2008). Positive density-dependence can occur when maternal plants provide their offspring with resources and shelter after dispersal; hence, they ameliorate environmental conditions and promote seedling establishment. This phenomenon is referred to as plant parental care (Wied and Galen 1998) and has been observed for some plant species living in stressful environments. For example, Barchuk *et al.* (2008) found a positive spatial association between juveniles and conspecific adults of *Aspidosperma quebracho-blanco* and proposed that one explanation to account for this pattern is that this species has recalcitrant seeds that do not form a seed bank; therefore, movement of seeds away from the maternal plant may imply a lower probability of success in finding a safe site for germination before dying of desiccation. The same principle may hold true for *M. coquimbensis* given its highly recalcitrant seeds. On the other hand, Wied and Galen (1998) argued that for plants to provide parental care, they require seeds to be retained near or within parental organs; consequently, limited seed dispersal or large dispersal units should promote the likelihood of maternal nursing. Again, *M. coquimbensis* is a species that fits both requirements, having with both large fruits and limited seed dispersal.

It is unknown if the initial fitness benefits derived by young *M. coquimbensis* plants under conspecifics persist through reproduction. These benefits may be offset if later growth is restricted due to competition or light limitation. Throughout its distribution range, however, *M. coquimbensis* individuals frequently grow aggregated, an observation that is not consistent with strong density-dependent mortality after establishment. Whether intraspecific facilitation is an idiosyncrasy of this species or a process to be expected in highly stressful environments is worthy of further study. The limited data available thus far (Wied and Galen 1998; Goldenheim *et al.* 2008; Fajardo and McIntire 2011; Cavieres and Penaloza 2012) suggest that facilitation among conspecifics is important for plant regeneration in these environments; however, the prevalence and the effect of intraspecific facilitation on population dynamics are still largely unknown.

## Conclusions

Evidence from this study reveals that, contrary to what would be expected under the Janzen–Connell recruitment model, plants can serve as nurses and facilitate growth and development of conspecific seedlings in stressful environments. Hence, these findings support the notion that in these environments intraspecific facilitation is a process that can promote plant regeneration, and override the effects of negative density-dependent processes during early establishment. Our results provide another example that reinforce previous assessments that population regulation in stressful environments can be a function of positive density-dependence. Finally, although conspecific plants are better nurses than rocks, these last elements may constitute the only solution for *M. coquimbensis* to establish in areas where the adult population has been extirpated, and should therefore not be disregarded in management practices as nurse elements to promote seedling recruitment.

## Sources of Funding

This research was supported by grants awarded to A.P.L. from the Rufford Small Grant Foundation, a FONDECYT Post-Doctoral Research Grant (3120123), a FONDECYT Initiation Grant (11140400), the Institute of Ecology and Biodiversity (IEB) Chilean Millennium Initiative (P05-002) and CONICYT (PFB-23). M.A.H.-M. was supported by the IEB (P05-002), and D.E.C. and P.G.-G. were supported by CONICYT Doctoral fellowships (21140050 and 21120854, respectively) and the IEB (P05-002).

## Contributions by the Authors

A.P.L., M.A.H.-M., D.E.C. and P.G.-G. performed the research and collected data. A.P.L. analysed all data and was the primary writer of the manuscript. F.A.S. and all other authors contributed to the study design, interpreted analysis results and contributed substantially to revisions.

## Conlficts of Interest

None declared.

## Supporting Information

The following additional information is available in the online version of this article—

**Appendix S1**. Detailed methods to model water retention curves for soil samples.

**Figure S1**. Water retention curves for soils of open areas, Myrcianthes coquimbensis and rock cavities.

**Figure S2**. Non-metric multidimensional scaling graph for soil chemistry of the three selected microhabitats: open interspaces, under conspecific shrubs and rock cavities.

## Supplementary Material

Supplementary-material
