# Supplementary material for: Conspecific plants are better ‘nurses’ than rocks: consistent results revealing intraspecific facilitation as a process that promotes establishment in a hyper-arid environment
Source: AoB Plants. 2017 Oct 24;9(6):plx056. doi: 10.1093/aobpla/plx056 (PMC5710651; doi:10.1093/aobpla/plx056)
Supplement: Supplementary-material [file plx056_suppl_supplementary-material.docx]

**SUPPORTING INFORMATION**

**Appendix 1**

To model water retention curves, we collected five soil samples from each microhabitat. Samples were placed in sealed plastic bags and immediately taken to the laboratory where they were oven-dried (Binder FED 53 - 720) for 48 hours at 70°C. Dried samples were individually weighed in using a scale (Scaltec SBC 31 electronic balance with a readability of 0.0001 g) and then saturated using a pipette; the amount of water added per sample varied between 5 – 7 ml. Samples were left at room temperature and sealed to prevent evaporation before measuring them. For the first measurement, we recorded the weight of each hydrated sample and then immediately its hydraulic potential (ψ_s_) with a WP4 Dewpoint PotentiaMeter. After measuring ψ_s_ samples were weighed again to record any water loss while measuring ψ_s_. Soil samples were then left to dry at room temperature for up to 12 hours until the next measurement. We conducted between nine and 13 measurements per sample to model the water retention curves. These were obtained by plotting the gravimetric water content (GWC) on the *x*-axis and ψ_s_ on the *y*-axis for each measurement. Individual curves were adjusted using a third-order inverse regression. Field capacity (FC) and the permanent wilting point (PWP) were then obtained from each curve.


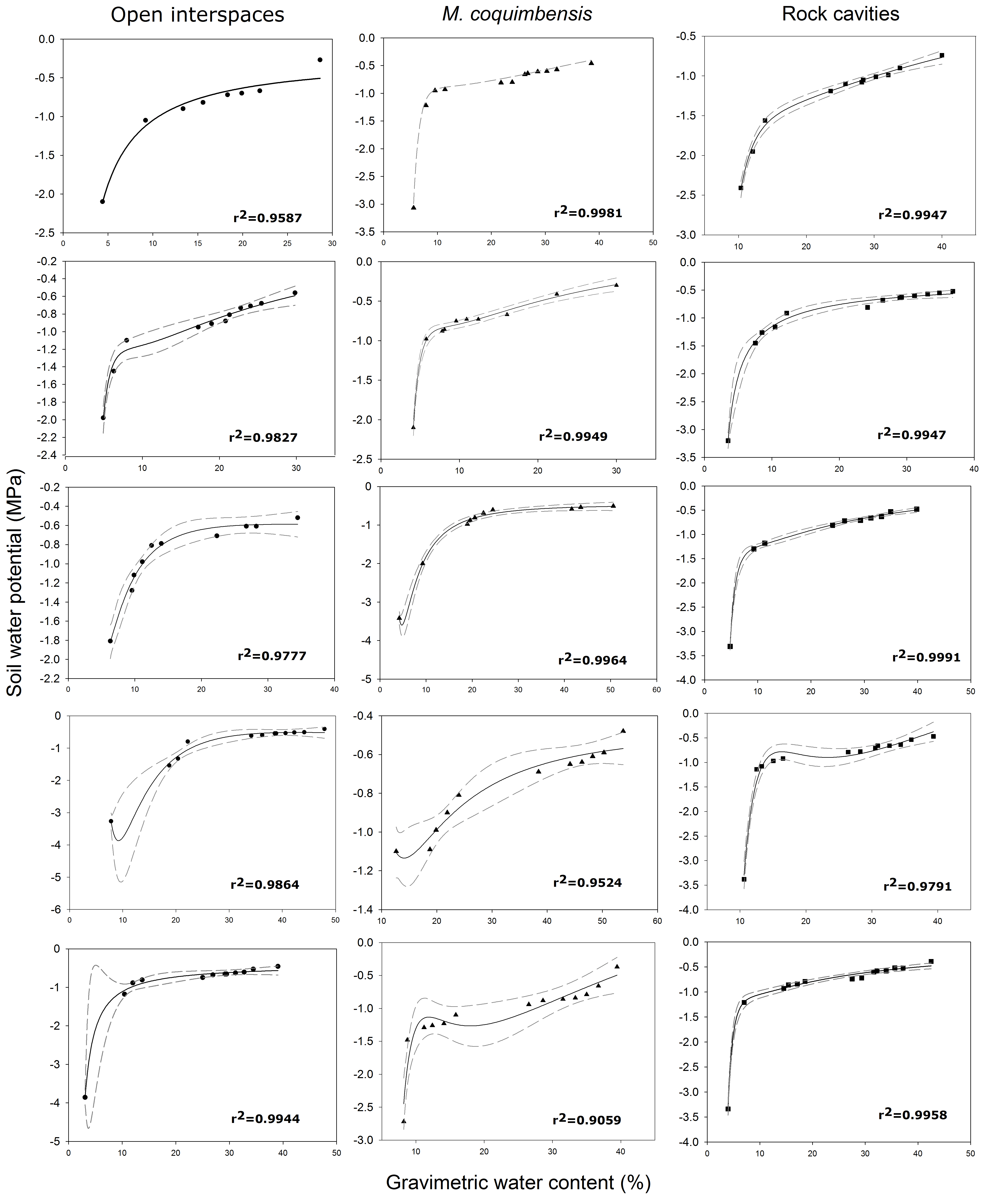


S1. Water retention curves for soils of open areas (solid circles), *M. coquimbensis* (solid triangles) and rock cavities (solid squares).


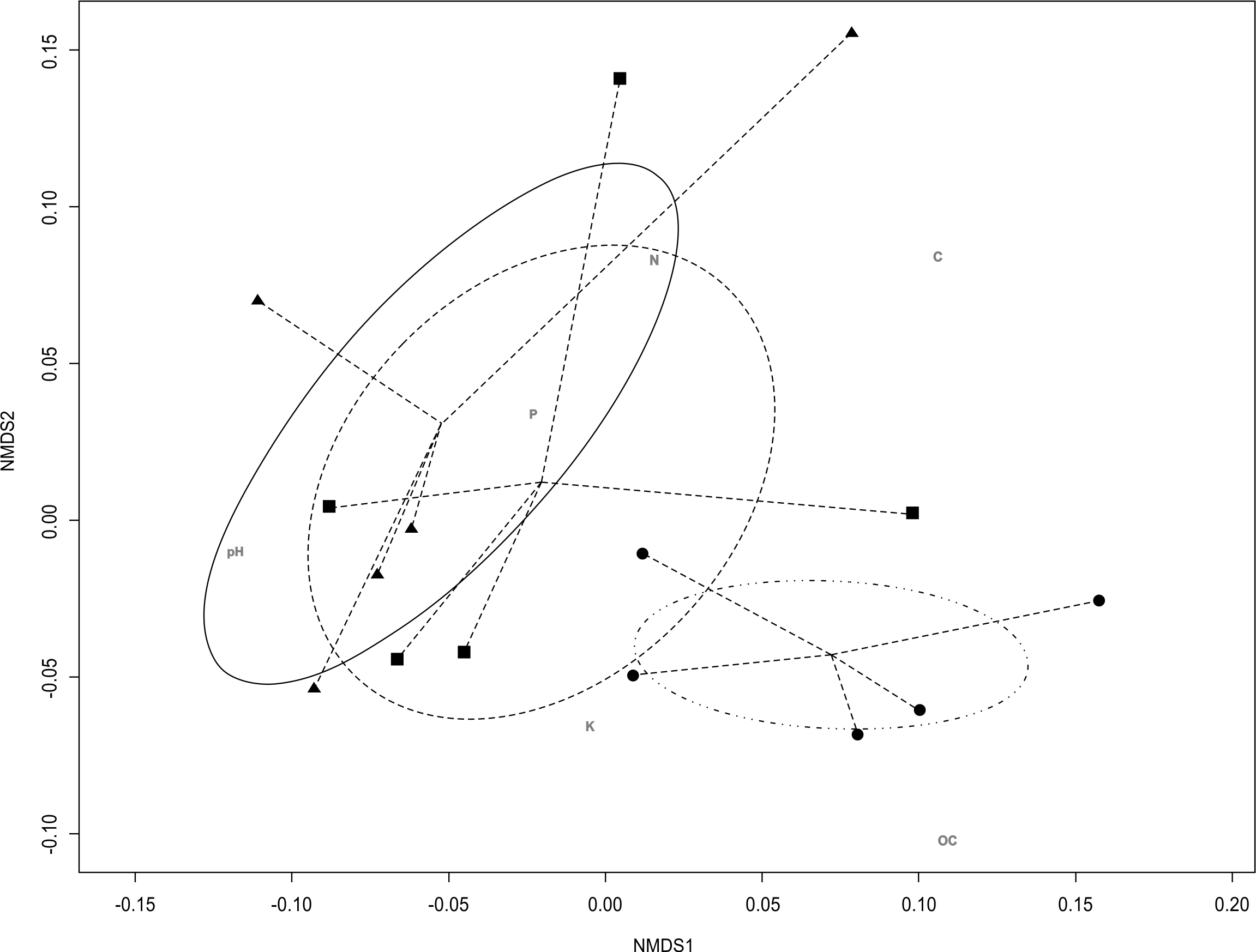


S2. Non-metric multidimensional scaling (NMDS) graph for soil chemistry of the three selected microhabitats: open inter-spaces (triangles; continuous ellipse), under conspecific shrubs (circles; dot-dash ellipse) and rock cavities (squares; dashed ellipse). Ellipses denote the dispersion around the mean values of each microhabitat. The individual points are the ordination of each line. The centroids are the average of the levels of the factors indicated. N: Nitrogen; P: Phosphorous; K: Potassium; C: conductivity; OC: Organic content and pH.
